# Metagenomics-Metabolomics Exploration of Three-Way-Crossbreeding Effects on Rumen to Provide Basis for Crossbreeding Improvement of Sheep Microbiome and Metabolome of Sheep

**DOI:** 10.3390/ani14152256

**Published:** 2024-08-03

**Authors:** Haibo Wang, Jinshun Zhan, Haoyun Jiang, Haobin Jia, Yue Pan, Xiaojun Zhong, Junhong Huo, Shengguo Zhao

**Affiliations:** 1 Jiangxi Province Key Laboratory of Animal Green and Healthy Breeding, Institute of Animal Husbandry and Veterinary, Jiangxi Academy of Agricultural Science, Nanchang 330200, China; wanghaibo8815@163.com (H.W.); zhanjinshun1985@163.com (J.Z.); jianghaoyun1995@163.com (H.J.); jiahaobin@jxaas.cn (H.J.); py13782525871@163.com (Y.P.); zhongcaoyangchu@163.com (X.Z.); 2 College of Animal Science and Technology, Gansu Agricultural University, Lanzhou 730070, China; 3 College of Animal Science and Veterinary Medicine, Tianjin Agricultural University, Tianjin 300384, China

**Keywords:** sheep, rumen, metagenomics, metabolomics, crossbreeding

## Abstract

**Simple Summary:**

Rumen microorganisms and their hosts have co-evolved over a long period of time, adapting to their environment. This co-evolution not only impacts the host phenotype but also establishes a sophisticated regulatory network with the host to uphold organismal homeostasis. This study’s findings indicated that the process of three-way hybridization had a notable impact on the taxonomic characteristics, functions, and metabolites of rumen microbes. Moreover, significant interactions were observed between rumen microbes and metabolites in sheep, with biomarkers playing a significant role in influencing the variation in rumen metabolites. Meanwhile, rumen microbial markers in Hu sheep exhibited a predominantly positive correlation with down-regulated metabolites and a negative correlation with up-regulated metabolites. In contrast, rumen microbial markers in CAH lambs showed an overall negative correlation with down-regulated metabolites and a positive correlation with up-regulated metabolites. The data obtained in this study provide a basis for understanding hybridization advantages from microbial metabolic pathways.

**Abstract:**

The objective of this experiment was to explore the effects of three-way hybridization on rumen microbes and metabolites in sheep using rumen metagenomics and metabolomics. Healthy Hu and CAH (Charolais × Australian White × Hu) male lambs of similar birth weight and age were selected for short-term fattening after intensive weaning to collect rumen fluid for sequencing. Rumen metagenomics diversity showed that Hu and CAH sheep were significantly segregated at the species, KEGG-enzyme, and CAZy-family levels. Moreover, the CAH significantly increased the ACE and Chao1 indices. Further, correlation analysis of the abundance of the top 80 revealed that the microorganisms were interrelated at the species, KEGG-enzyme, and CAZy-family levels. Overall, the microbiome significantly affected metabolites of the top five pathways, with the strongest correlation found with succinic acid. Meanwhile, species-level microbial markers significantly affected rumen differential metabolites. In addition, rumen microbial markers in Hu sheep were overall positively correlated with down-regulated metabolites and negatively correlated with up-regulated metabolites. In contrast, rumen microbial markers in CAH lambs were overall negatively correlated with down-regulated metabolites and positively correlated with up-regulated metabolites. These results suggest that three-way crossbreeding significantly affects rumen microbial community and metabolite composition, and that significant interactions exist between rumen microbes and metabolites.

## 1. Introduction

Ruminants can utilize plant feeds (i.e., straw, hay, and grass) to provide high-quality meat protein for human needs. Mutton is favored by consumers due to its unique flavor, nutrient richness, and low cholesterol content [1,2,3]. Hu sheep, recognized for its four-season estrus cycle, early onset of sexual maturity, and high litter productivity, is classified as one of China’s indigenous first-class protected breeds. However, its primary drawback lies in its low meat yield [4]. Meat yield represents a critical economic trait for livestock, and enhancing growth performance is essential for improving farm economic efficiency [5,6]. Crossbreeding offers more significant genetic gains compared to breed selection for improving growth performance [6,7,8]. Therefore, in sheep production practice, crossbreeding introduced breeds with local breeds is commonly used to enhance offspring productivity or develop new breeds [6,9,10]. Furthermore, there is an intricate regulatory network that interplays among body weight, host genetics, and rumen microbes in sheep. Research results indicate that the heritability of body weight in Hu sheep at 180 days of age is 39%, with rumen microorganisms contributing 20% of the phenotypic variance when considering host genetics [11].

The rumen of ruminants has the capacity to ferment plant feeds into small molecular compounds that function as an energy source for the animal. This capability is a result of the synergistic evolution of the rumen microbes and the host, which develops over a prolonged period of adaptation to the environment [12]. In this scenario, the host creates a stable internal environment for the microorganisms, which results in microbial metabolism producing small-molecule compounds that can regulate host physiological functions and influence host traits [11,13,14,15], including involvement in fat deposition [16], body weight [11], feed efficiency [17,18], and marbling grade [19]. The most extensive interactions between rumen microbes and their hosts are realized through the consumption and production of metabolites, especially small-molecule compounds such as short-chain fatty acids [20,21]. Moreover, rumen metabolites have been shown to be influenced by genetic, environmental microbiome, and dietary factors [22,23]. In addition, ruminant rumen microbes are structurally similar [24], but they are involved in the regulation of host physiological functions in which host genes and phenotypes form a complex regulatory network [12,17,19,25], and host genetics play an important role in the formation of rumen microbial communities, including heterozygosity arising from parental gene interactions, which can influence rumen microbial composition [26,27].

It has been found that crossbreeding can significantly improve the productive performance of offspring [6,8,9,10]. In practice, Hu sheep are usually used as the female parent for breeding new strains and varieties [28,29]. A previous study discovered that three-way-hybrid sheep can modulate rumen parameters by altering the microbiota structure, which affects serum physiological indices and enhances animal performance [8]. This hybrid also significantly improved lamb meat quality in terms of crude protein, essential amino acids, and non-essential amino acids [30]. Metabolomics analysis revealed that amino acid metabolism, particularly arginine and proline metabolism, plays a crucial role in regulating the meat quality of hybrid progeny [6]. Therefore, it is hypothesized that host genetics may enhance sheep growth and development by influencing rumen microbial community structure and rumen metabolism. In this study, we investigated whether three-way crossbreeding can alter rumen microbial communities and rumen metabolism through metagenomics and metabolomics studies and whether there is a regulatory relationship between them. To test this hypothesis, a specific Charolais ram was used as a terminal ram to cross ewes with Australian White × Hu genetics to produce a Charolais × Australian White × Hu (CAH), which was then compared with pure native Hu lambs. Rumen macrogenomics and metabolomics were employed to study the effects of three-way crossbreeding on the rumen community and metabolism in sheep while maintaining a consistent feeding environment and excluding the influence of other factors.

## 2. Materials and Methods

The experimental design and husbandry management adhered to the guidelines set by the ethical committee of the Institute of Animal Husbandry and Veterinary Medicine, Jiangxi Academy of Agricultural Sciences, approved under (2010−JAAS−XM−01). The animal experiments were conducted at the scientific and technological service workstation of the Jiangxi Academy of Agricultural Sciences (Ganzhou Lvlinwan Agricultural and Animal Husbandry Co., Ltd., Ganzhou, China).

### 2.1. Experimental Animals

Healthy male lambs with similar birth weights from Hu (Hu × Hu) and CAH (Charolais × Australian White × Hu) breeds were weaned centrally and uniformly after being nursed by their mothers until 45 days of age. Subsequently, 11 CAH and 11 Hu lambs were selected for 90 days of short-term fattening. All CAH and Hu lamb were centrally allocated to group pens. The feeding regimen involved providing feed at 8:00 am and 17:00 pm, with lambs having ad libitum access to feed and water. Immunization protocols were implemented in compliance with company policies. The diet consisted of digestible energy at 10.82 MJ/kg, crude protein at 16.95%, neutral detergent fiber at 27.68%, acid detergent fiber at 17.31%, calcium at 0.65%, and phosphorus at 0.35% for the total mixed ration. Detailed nutrient fractions are described in Wang et al. [8].

### 2.2. Sample Collection

Rumen fluid was collected from lambs using a gastric tube rumen sampler in the morning prior to feeding at 135 days of age in all lambs, following the method described in detail by Wang et al. [8]. The rumen fluid was dispensed into cryopreservation tubes, snap frozen in liquid nitrogen, and sent to the laboratory to be stored at −80 °C for subsequent macrogenomic and metabolomic sequencing. Body size correlation was measured using a soft ruler and an electronic scale. Ruminal enzyme activities were determined according to the kit instructions (Shanghai Kexing Biotechnology Co., Ltd., Shanghai, China). Ruminal VFA molar concentration was determined by gas chromatography (GC-7890B, Agilent Technologies, Petaling Jaya, Malaysia). Rumen pH was determined using a portable pH meter (PHBJ-260F; INESA Scientific Instruments Co., Ltd., Shanghai, China). Subsequently, acetate/propionate (A/P) and VFA molar ratios were calculated. Detailed information on the experimentally relevant data is described in the pre-publication article by Wang et al. [8].

### 2.3. Metagenome Sequencing and Bioinformatics Analysis

Bacterial DNA was extracted from the rumen fluid of 20 lambs following the instructions of the TGuide S96 Magnetic Stool DNA Kit from Beijing. Initially, the bacterial genomic DNA suitable for testing was enzymatically fragmented. Subsequently, the fragmented DNA underwent end repair, adaptor ligation, library amplification, and purification to produce a qualified library. The Qsep-400 was utilized for fragment quality control, and sequencing of the qualified libraries was carried out using the Illumina NovaSeq6000 (Illumina Inc., San Diego, CA, USA). Raw tags were filtered using fastp (version 0.23.1) software [31] and compared with the host genome sequence using bowtie2 (version 2.2.4) [32] to eliminate host contamination and obtain high-quality clean tags. Following this, macrogenome assembly was conducted using the MEGAHIT (version 1.1.2) [33] software to filter contig sequences shorter than 300 bp, and the assembly results were assessed using the QUAST (version 2.3) software [34]. Redundancy was then eliminated using the MMseqs2 (https://github.com/soedinglab/mmseqs2, Version 12-113e3, 2 October 2023) software [35] based on set thresholds (95% similarity and 90% coverage). Simultaneously, the annotation process identified carbohydrate-active enzymes in the genome by comparing the protein sequences of non-redundant genes to the CAZy [36] database separately. Subsequently, representative sequences from the non-redundant gene catalog were aligned with the Nr [37] (Non-Redundant Protein Database) and KEGG [38] (Kyoto Encyclopedia of Genes and Genomes) databases using BLASTP (expected e-value is 10^−5^) to obtain annotation information.

### 2.4. Metabolome Sequencing and Bioinformatics Analysis

An amount of 100 μL of the sample was mixed with 500 μL of extraction solution containing an internal standard (V_methanol_:V_acetonitrile_ = 1:1, internal standard concentration 20 mg/L); this was vortexed and mixed for 30 s. Next, following ultrasonic ice bath (10 min), standing (1 h, −20 °C), and centrifugation (15 min, 4 °C, 12,000 rpm), 500 μL of the supernatant was taken to dry the extract in a vacuum concentrator. Subsequently, the extract was reconstituted (160 μL, V_acetonitrile_:V_water_ = 1:1), vortexed (30 s), subjected to ultrasonic ice bath (10 min), and centrifuged (15 min, 4 °C, 12,000 rpm), and then 120 μL of the supernatant was taken in a 2 mL injection vial and assayed on the machine (10 μL of each sample was taken and mixed to form a QC sample). The LC/MS system for metabolomics analysis is composed of Waters Acquity I-Class PLUS ultra-high-performance liquid tandem Waters Xevo G2-XS QTOF high-resolution mass spectrometer (Waters, Milford, MA, USA) (Waters Acquity UPLC HSS T3, 1.8 μm, 2.1 × 100 mm). Subsequently, mass spectral data were collected with a high-resolution mass spectrometer (Waters Xevo G2-XS QTOF) in MSe mode under the control of the acquisition software (MassLynx V4.2, Waters). Finally, Progenesis QI (version 4.0) software was used for peak extraction, peak comparison, and identification of the raw data obtained using the online METLIN database and Biomark’s own libraries [39]. Metabolites were screened (FC > 1, *p* value < 0.05, and VIP > 1) and annotated using the KEGG (https://www.genome.jp/kegg/, accessed on 30 September 2023) database, and then the annotated metabolites were mapped to the KEGG pathway database [40,41].

### 2.5. Data Statistics and Analysis

The alpha diversity of the experimental sample was assessed through the QIIME2 (https://qiime2.org, accessed on 6 June 2020) software, which including ACE, Chao1, Shannon, and Simpson indices. The degree of similarity of the sample microbial communities was evaluated based on the binary Jaccard distance using beta diversity, which primarily involved principal coordinate analysis (PCoA) and non-metric multidimensional scaling (NMDS). Additionally, a Student’s t-test was conducted to determine if there were significant differences in alpha and beta diversity between different sample groups. Linear discriminant analysis (LDA) effect size (LEfSe [42]) was utilized to identify significant taxonomic differences between groups. Furthermore, orthogonal projections to latent structures discriminant analysis (OPLS-DA, version 1.6.2 [43]) was employed to identify metabolic differences between the two groups.

## 3. Results

### 3.1. Genome Profiling of Rumen Microorganisms

#### 3.1.1. Sequencing and Diversity Analysis of Rumen Microbiota

**Figure 1:**
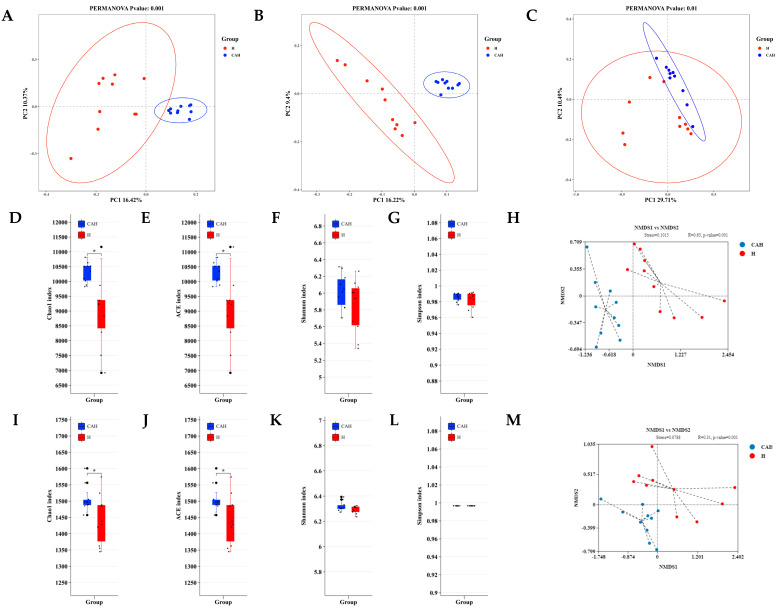

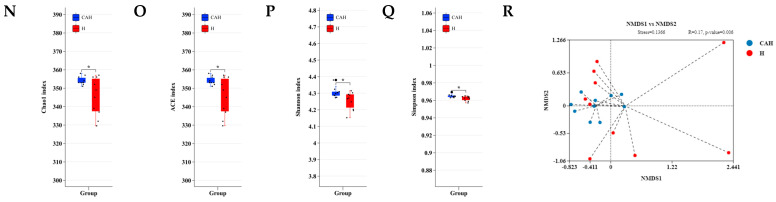
Comparison of rumen microbial diversity in Hu and CAH lambs. Figures (**A**–**C**) show PCoA results for archaea (**A**), bacteria (**B**), and fungi (**C**) at the species level. Next, alpha diversity analysis of Hu and CAH rumen microbes at species (**D**–**G**), KEGG-enzyme (**I**–**L**) and CAZy-family levels (**N**–**Q**) were presented. Finally, Hu and CAH lamb were analyzed by non-metric multidimensional scaling (NMDS) at species (**H**), KEGG-enzyme (**M**), and CAZy-family levels (**R**). * in the alpha diversity plot indicates *p* < 0.05.

Metagenomic analysis of rumen contents in Hu and CAH showed that raw reads were obtained, excluding low-quality and n-containing reads. Hu and CAH acquired 7,145,045,673 and 6,617,688,378 clean databases, respectively. Furthermore, optimized clean no-host databases were obtained for subsequent analysis after removing the host genome sequence, resulting in 7,062,137,125 and 6,538,847,179 clean databases for Hu and CAH, respectively. Finally, Hu and CAH obtained 47,197,640 and 43,688,879 effective reads, respectively. This experiment focuses on the rumen microbiome and metabolome to investigate potential mechanisms for improving growth performance in three-way-crossbred lambs. Therefore, our subsequent analyses focused mainly on the archaeal, bacterial, and fungi species. According to the principal coordinate analysis (PCoA) at the species level, archaeal (Figure 1A), bacterial (Figure 1B), and fungal (Figure 1C) species showed significant differences in both groups (*p* < 0.05).

According to the rumen community analyzed in Figure 1A, at the species and KEGG-enzyme levels, it can be seen that Hu lambs were significantly lower than CAH lambs in Chao1 (Figure 1D,I) and ACE (Figure 1E,J) indices (*p* < 0.05). However, there was no significant difference in either the Shannon (Figure 1F,K) or Simpson (Figure 1G,L) indices (*p* > 0.05). Moreover, at the CAZy-family level, the Chao1 (Figure 1N), ACE (Figure 1O), Shannon (Figure 1P), and Simpson (Figure 1Q) indices of Hu lambs were significantly lower than those of CAH lambs (*p* < 0.05). Further, the results analyzed in the NMDS and analysis of similarity (ANOSIM) of rumen microbes of Hu and CAH lambs showed stress values of less than 0.2 and *p* value values of less than 0.05. This result suggests that there are significant differences between the microbes of Hu and CAH lambs at the species (stress = 0.1015, R = 0.65, *p* = 0.001, Figure 1H), KEGG-enzyme (stress = 0.0788, R = 0.31, *p* =0.001, Figure 1M), and CAZy-family levels (stress = 0.1366, R = 0.17, *p* = 0.006, Figure 1R).

#### 3.1.2. Analysis of Rumen Microbial Composition, Function, and Correlation

**Figure 2:**
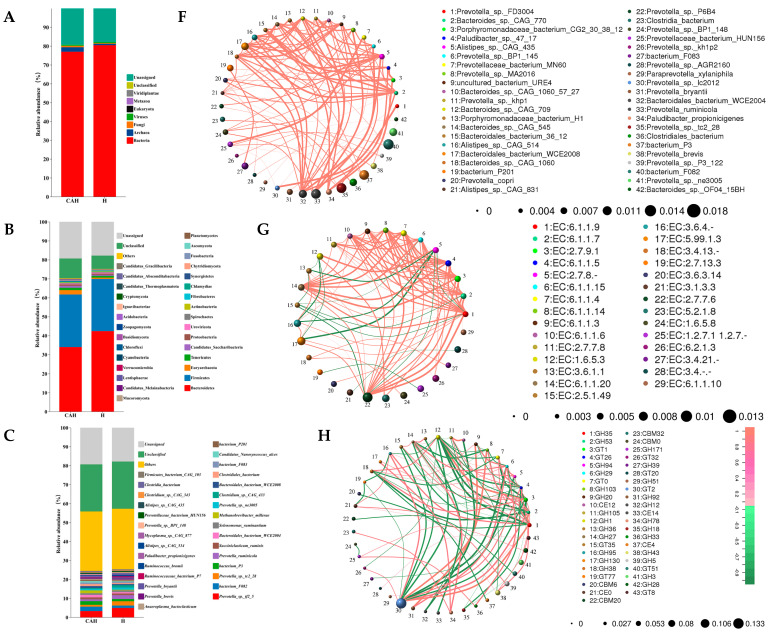

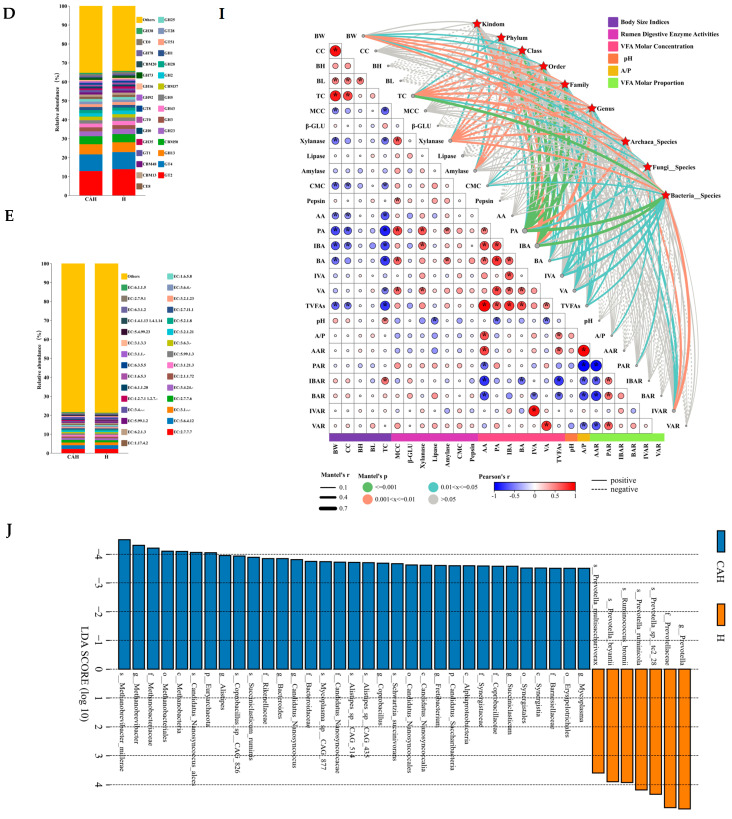
Analysis of rumen microbial composition, function, and correlation. Stacked histograms of the relative abundance of the rumen microbiota of the Hu and CAH groups at the kingdom (**A**), phylum (**B**), species (**C**), CAZy−family (**D**), and KEGG−enzyme (**E**) levels of the top 30. In addition, correlation network maps were constructed with abundance top 80, *p* < 0.05, at the species (**F**), KEGG−enzyme (**G**), and CAZy−family (**H**) levels, respectively. In the network diagram, the size of the node circle represents abundance, the connecting line of the node represents correlation, the thickness of the line indicates the strength of the correlation, red indicates positive correlation, and green indicates negative correlation. We further correlated rumen microorganisms with sheep body size indicators and rumen parameters (**I**). Pairwise comparisons of body size indicators (BW: body weight, BL: body length, CC: chest circumference, BH: body height, and TC: tube circumference), rumen enzyme activity (MMC, β−GLU, xylanase, lipase, amylase, CMC, and pepsin), rumen VFA molar concentration (AA: acetic acid, PA: propionic acid, BA: butyric acid, IBA: isobutyric acid, VA: valeric acid, IVA: isovaleric acid, and TVFAs), pH, A/P (acetic acid/propionic acid), and molar proportion (AAR: AA ratio, PAR: PA ratio, BAR: BA ratio, IBAR: IBA ratio, VAR: VA ratio, and IVAR: IVA ratio) are shown Figure 2. Spearman’s correlation coefficient is indicated by the colored circles. Edge width corresponds to the Mantel’s r statistic for the corresponding distance correlations, and edge color denotes the statistical significance based on permutations. In the heat map, * indicates significant correlation, and values indicate correlation coefficients. Finally, the linear discriminant analysis effect size (LEfSe) analysis of sheep rumen microorganisms is shown in (**J**).

Differential analysis revealed that there were a total of 3552 distinct microorganisms in archaea, bacteria, and fungi. Microbiome analysis revealed 121 (CAH was up-regulated by 101 and down-regulated by 20), 73 (CAH was up-regulated by 60 and down-regulated by 13), and 3361 (CAH was up-regulated by 2581 and down-regulated by 780) significantly different archaea, fungi, and bacteria in the rumen of Hu and CAH lambs, respectively (*p* < 0.05). The dominant microorganisms in the rumen are bacteria (H: 80.45%, CAH: 76.98%) (Figure 2A). Additionally, the dominant microorganisms in the rumen at the phyla level were Bacteroidetes (H: 42.32%, CAH: 33.92%), Firmicutes (H: 27.54%, CAH: 27.75%), Euryarchaeota (H:0.71%, CAH: 2.28%) and Tenericutes (H: 0.93%, CAH: 1.25%) (Figure 2B).

First, according to the comparative analysis, phyla-level top 30 differences revealed that the abundance of 13 phyla (Euryarchaeota, Tenericutes, Candidatus Saccharibacteria, Synergistetes, Planctomycetes, Lentisphaerae, Verrucomicrobia, Cyanobacteria, Chloroflexi, Acidobacteria, Candidatus Thermoplasmatota, Candidatus Absconditabacteria, and Candidatus Gracilibacteria) in CAH was higher than that in Hu (*p* < 0.05), and two phyla (Bacteroidetes and Fusobacteria) were significantly lower than those in the Hu group (*p* < 0.05) (Figure 2B). At the species level (top 30), compared to Hu sheep, CAH significantly increased *bacterium_F082*, *bacterium_P3*, *Bacteroidales_bacterium_WCE2004*, *Methanobrevibacter_millerae*, *Bacteroidales_bacterium_WCE2008*, *Clostridiales_bacterium*, *bacterium_F083*, *Candidatus_Nanosyncoccus_alces*, *bacterium_P201*, *Alistipes_sp._CAG_51*, *Mycoplasma_sp._CAG_877*, and *Alistipes_sp._CAG_435* but decreased *Prevotella_sp._tc2_28*, *Prevotella_ruminicola*, *Prevotella_sp._ne3005*, *Prevotella_brevis*, *Prevotella_bryantii*, *Ruminococcaceae_bacterium_P7*, *Prevotella_sp._BP1_148*, and *Prevotellaceae_bacterium_HUN156* (*p* < 0.05) (Figure 2C). At the CAZy-family level (top 30), compared to Hu sheep, CAH significantly increased GH13, GH3, GH28, GH1, GT1, GH92, and GH38 but decreased GT2, GH23, GH43, GH2, GH35, GT0, and GH73 (*p* < 0.05) (Figure 2D). At the KEGG-enzyme level (top 30), compared to Hu sheep, CAH significantly increased EC:2.7.7.6 (DNA-directed RNA polymerase), EC:5.99.1.3 (DNA topoisomerase- ATP-hydrolysing), EC:3.6.3.- (catalyzing transmembrane movement of substances),EC:3.6.4.- (cellular and subcellular movement), EC:6.2.1.3 (long-chain-fatty-acid-CoA ligase), EC:1.2.7.1 1.2.7.- (pyruvate-ferredoxin/flavodoxin oxidoreductase), EC:6.1.1.20 (phenylalanine-tRNA ligase), EC:3.1.1.- (Carboxylic ester hydrolases), EC:2.7.9.1 (pyruvate, phosphate dikinase), and EC:6.1.1.5 (isoleucine-tRNA ligase) but decreased EC:2.1.1.72 (site-specific DNA-methyltransferase), EC:3.1.21.3 (type I site-specific deoxyribonuclease), EC:5.2.1.8 (peptidylprolyl isomerase), EC:3.2.1.23 (beta-galactosidase), EC:1.6.5.3 (NADH:ubiquinone reductase- H^+^-translocating), and EC:1.4.1.13 1.4.1.14 (glutamate synthase) (*p* < 0.05) (Figure 2E).

Then, we performed correlation analyses differences at the species (Figure 2F), KEGG-enzyme (Figure 2G), and CAZy-family (Figure 2H) levels (top 80), respectively. At the species level, *Prevotella_sp._FD3004* and *Prevotella_sp._lc2012* were maximally positively correlated (Figure 2F). Additionally, there was a positive correlation among species, with *Bacteroides_sp._CAG_1060* showing the closest correlation with the other species (Figure 2F). The top five differentially correlated species at the species level were *bacterium_F082*, *bacterium_P3*, *Prevotella_sp._tc2_28*, *Prevotella_ruminicola*, and *Bacteroidales_bacterium_WCE2004* (Figure 2F). At the KEGG-enzyme level, EC:6.1.1.9 (valine-tRNA ligase) and EC:2.7.7.6 (DNA-directed RNA polymerase) were maximally positively correlated (Figure 2G). EC:6.1.1.9 (valine-tRNA ligase) was most closely correlated with the other species, and the negative correlations were mainly concentrated in EC:2.7.8.- (Transferases for other substituted phosphate groups) and EC:2.5.1.49 (O-acetylhomoserine aminocarboxypropyltransferase) (Figure 2G). At the KEGG-enzyme level, the top five differentially correlated abundances were EC:2.7.7.6 (DNA-directed RNA polymerase), EC:5.99.1.3 (DNA topoisomerase- ATP-hydrolysing), EC:5.2.1.8 (peptidylprolyl isomerase), EC:3.6.4.- (cellular and subcellular movement), and EC:1.6.5.8 (NADH: ubiquinone reductase -Na^+^-transporting) (Figure 2G). At the CAZy-family level, GH35 and GH1 were maximally negative correlated (Figure 2H). GH35 and GH1 were most closely correlated with the other species (Figure 2H). At the CAZy-family level, the top five differentially correlated abundances were GT2, GH43, GH28, GH1, and GT51 (Figure 2H).

Subsequently, we further correlated rumen microorganisms with sheep body size indicators, rumen enzyme activity, rumen VFA molar concentration, and molar proportion, as shown in Figure 2I. The correlations between the body size indicators of sheep were mainly positive, and microorganisms significantly affected BW and TC, with the most significant correlations between BW and CC (Figure 2I). Additionally, there was an overall positive correlation between rumen fluid enzyme activities in sheep, but β-GLU was negatively correlated with CMC and amylase. Meanwhile, MCC showed significant positive correlation with xylanase and pepsin, respectively. Microorganisms had significant effects on xylanase, amylase, and CMC but not on MCC, β-GLU, and pepsin (Figure 2I). Ruminal pH showed an overall negative correlation with VFA molar concentration, but there was an overall significant positive correlation between the VFAs, and the microorganisms significantly influenced the VFA molar concentration (PA, IBA, BA, IVA, VA, and TVFAs) (Figure 2I). Additionally, A/P showed a significant positive correlation with AAR, but it was significantly negatively correlated with PAR, IBAR, BAR, and VAR (Figure 2I). Meanwhile, AAR showed a significant negative correlation with VFA molar proportion (PAR, IBAR, BAR, and VAR), while other VFA molar proportions showed a basically positive correlation with each other (Figure 2I). Among the phenotypes associated with body size indicators and rumen parameters, rumen microbes had the highest correlation with PA, followed by IBA (Figure 2I).

Finally, LEfSe was used to analyze biomarkers in the rumen of crossbred offspring lambs, with an LDA score of 3.5. The analysis revealed 34 statistically different biomarkers in the rumen fluid of lambs from various crossbreeding offspring, including 7 in Hu and 33 in CAH. At the species level, five biomarkers were found to be *Prevotella_sp__tc2_28*, *Prevotella_ruminicola*, *Ruminococcus_bromii*, *Prevotella_bryantii*, and *Prevotella_multisaccharivorax* in the Hu group. The eight biomarkers in CAH sheep were *Schwartzia_succinivorans*, *Alistipes_sp__CAG_435*, *Alistipes_sp__CAG_514*, *Mycoplasma_sp__CAG_877*, *Succiniclasticum_ruminis*, *Coprobacillus_sp__CAG_826*, *Candidatus_Nanosyncoccus_alces*, and *Methanobrevibacter_millerae* (Figure 2J).

### 3.2. Comparison of Ruminal Metabolites in Hu and CAH Lambs

**Figure 3:**
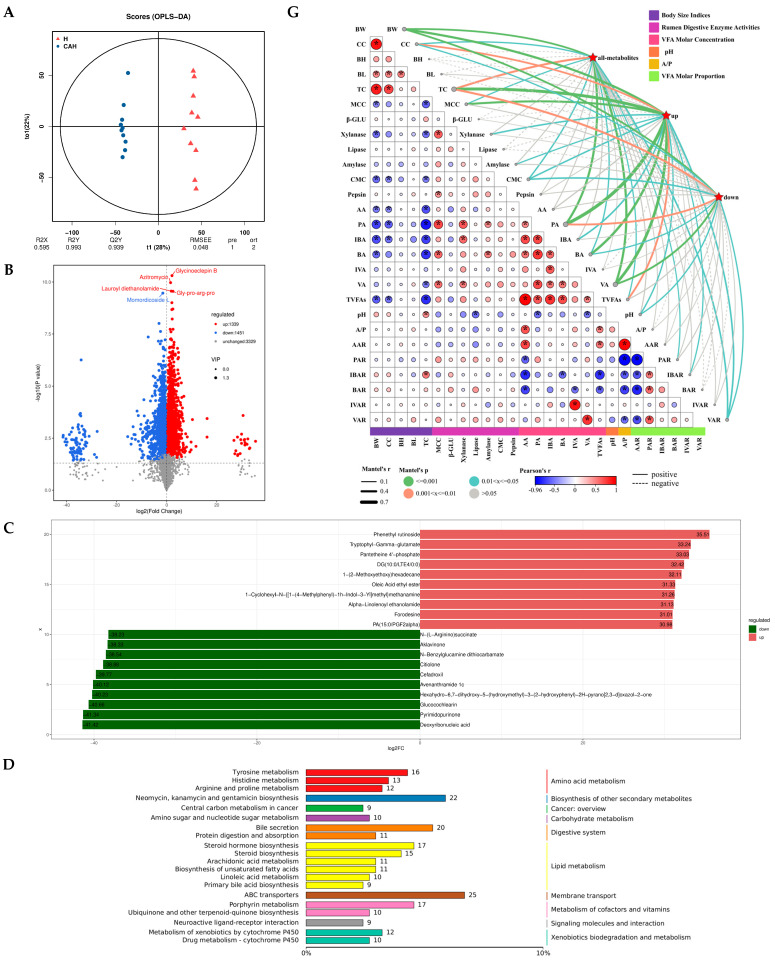

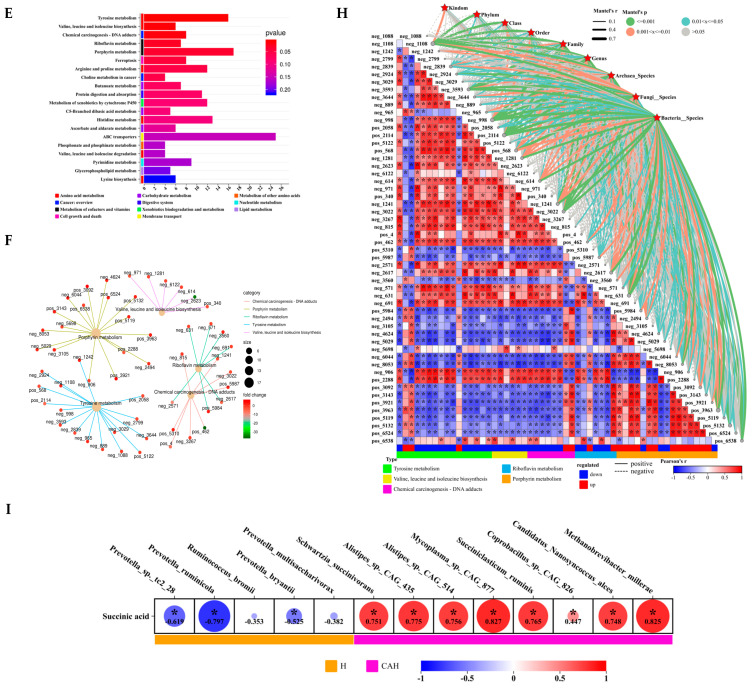
Comparison of ruminal metabolites in Hu and CAH lambs. The OPLS-DA model score plots for the Hu and CAH groups are displayed in (**A**), and the metabolite volcano plots are shown in (**B**). In addition, the top 10 with the largest up-and down-regulation of differential metabolite multiplicity are shown in (**C**). Meanwhile, (**D**) plots the information of the top 20 entries with the largest number of differential metabolites annotated to the differential metabolites in the pathway using the KEGG database annotation. Subsequently, the metabolic pathways of top 20 were further mapped (**E**), and the KEGG enrichment network map of the top 5 differential metabolites was constructed (**F**). Meanwhile, we further correlated rumen metabolites with sheep body size indexes and rumen parameters (**G**), as well as rumen microbes and differential metabolites (**H**) in the top 5 pathways of the KEGG enrichment network map. Pairwise comparisons of body size indicators and rumen parameters are shown (**G**), whereas pairwise comparisons of differential metabolites in the top 5 pathways of the KEGG enrichment network maps are shown in (**H**). The correlation of rumen differential biomarkers with succinic acid is shown in (**I**). Spearman’s correlation coefficient is indicated by the colored circles. Edge width corresponds to the Mantel’s r statistic for the corresponding distance correlations, and edge color denotes the statistical significance based on permutations. In the heat map, * indicates significant correlation, and values indicate correlation coefficients.

The OPLS-DA from Figure 3A indicated that Hu lamb and CAH lamb rumen fluid samples were distinguished. Moreover, the evaluated models were stable and reliable (Q2Y = 0.939 > 0.9) for screening differential metabolites for subsequent analysis. With VIP > 1 and *p* < 0.05 as the screening criteria for differentially accumulated metabolites, 2970 (1339 up-regulated and 1451 down-regulated by CAH vs. Hu lambs) differentially accumulating metabolites were screened from a total of 6119 metabolites (Figure 3B). Figure 3C demonstrates the significant up-regulation (Phenethyl rutinoside, Tryptophyl-Gamma-glutamate, Pantetheine 4’-phosphate, DG(10:0/LTE4/0:0), 1-(2-Methoxyethoxy)hexadecane, Oleic Acid ethyl ester, 1-Cyclohexyl-N-([1-(4-Methylphenyl)-1h-Indol-3-Yl]methyl)methanamine, Alpha-Linolenoyl ethanolamide, Forodesine, and PA(15:0/PGF2alpha)) and down-regulation (Deoxyribonucleic acid, Pyrimidopurinone, Glucocochlearin, Hexahydro-6,7-dihydroxy-5-(hydroxymethyl)-3-(2-hydroxyphenyl)-2H-pyrano [2,3-d]oxazol-2-one, Avenanthramide 1c, Cefadroxil, Citiolone, N-Benzylglucamine dithiocarbamate, Aklavinone, and N-(L-Arginino)succinate) of CAH vs. Hu top 10 metabolites.

Subsequently, we annotated the differential metabolites using the KEGG database and selected the top 20 with the most annotations to differential metabolites in the pathway, as shown in Figure 3D, including amino acid metabolism (3 pathway, including tyrosine metabolism, histidine metabolism, and arginine and proline metabolism), biosynthesis of other secondary metabolites (1 pathway, including neomycin, kanamycin, and gentamicin biosynthesis), cancer: overview (1 pathway, including central carbon metabolism in cancer), carbohydrate metabolism (1 pathway, including amino sugar and nucleotide sugar metabolism), digestive system (2 pathway, including bile secretion and protein digestion and absorption), lipid metabolism (6 pathway, including steroid hormone biosynthesis, steroid biosynthesis, arachidonic acid metabolism, biosynthesis of unsaturated fatty acids, linoleic acid metabolism, and primary bile acid biosynthesis), membrane transport (1 pathway, including ABC transporters), the metabolism of cofactors and vitamins (2 pathway, including porphyrin metabolism, ubiquinone, and other terpenoid-quinone biosynthesis), signaling molecules and interaction (1 pathway, including neuroactive ligand–receptor interaction), and xenobiotics biodegradation and metabolism (2 pathway, including the metabolism of xenobiotics by cytochrome P450 and drug metabolism-cytochrome P450).

Then, we further analyzed the pathways, as shown in Figure 3E, and constructed top five differential metabolite KEGG enrichment network graphs (Figure 3F). The study of the biological process of the KEGG pathway enriched by these differential metabolites revealed that they were mainly involved in biological processes such as amino acid metabolism (tyrosine metabolism and valine, leucine, and isoleucine biosynthesis), cancer: overview (chemical carcinogenesis-DNA adducts), and the metabolism of cofactors and vitamins (riboflavin metabolism and porphyrin metabolism) (Figure 3E,F).

Finally, the correlation analysis of metabolites with body size indexes and rumen parameters of sheep in Figure 3G showed that metabolites significantly affected body size indexes (BW, CC, and TC), rumen enzyme activity (MCC, xylanase, amylase, and CMC), rumen pH, rumen VFA molar concentration (PA, IBA, BA, VA, and TVFAs), and molar proportion (PAR, IBAR, and VAR) (Figure 3G). Additionally, in Figure 3H, we analyzed the differential metabolites in the top five pathway in the constructed KEGG enrichment network map for correlation with the microbiome. Overall, the microbiome significantly affected the metabolites in the top five pathway, with the strongest correlation with neg_1108 (succinic acid of the tyrosine metabolism pathway), followed by pos_3963 (urobilinogen of the porphyrin metabolism pathway), neg_998 (dopaquinone of the tyrosine metabolism pathway), pos_2114 (leucodopachrome of the tyrosine metabolism pathway), and neg_631 (D-Ribulose 5-phosphate of the riboflavin metabolism pathway), respectively (Figure 3H). Meanwhile, the differential metabolites were also significantly correlated with each other. Among them, the differential metabolites in the tyrosine metabolism, chemical carcinogenesis-DNA adducts, riboflavin metabolism, and valine, leucine, and isoleucine biosynthesis pathways, in general, were significantly negatively correlated with those in the porphyrin metabolism pathway, respectively (Figure 3H). Subsequently, we further showed by correlation analysis of succinic acid with species-level microbial markers that *Prevotella_sp._tc2_28*, *Prevotella_ruminicola*, and *Prevotella_bryantii* were significantly negatively correlated and associated with succinic acid, but *Schwartzia_succinivorans*, *Alistipes_sp._CAG_435*, *Alistipes_sp._CAG_514*, *Mycoplasma_sp._CAG_877*, *Succiniclasticum_ruminis*, *Coprobacillus_sp._CAG_826*, *Candidatus_Nanosyncoccus_alces*, and *Methanobrevibacter_millerae* showed significant positive correlation with succinic acid (Figure 3I).

### 3.3. Relationship between Rumen Biomarkers and Differential Metabolites in Sheep

**Figure 4:**
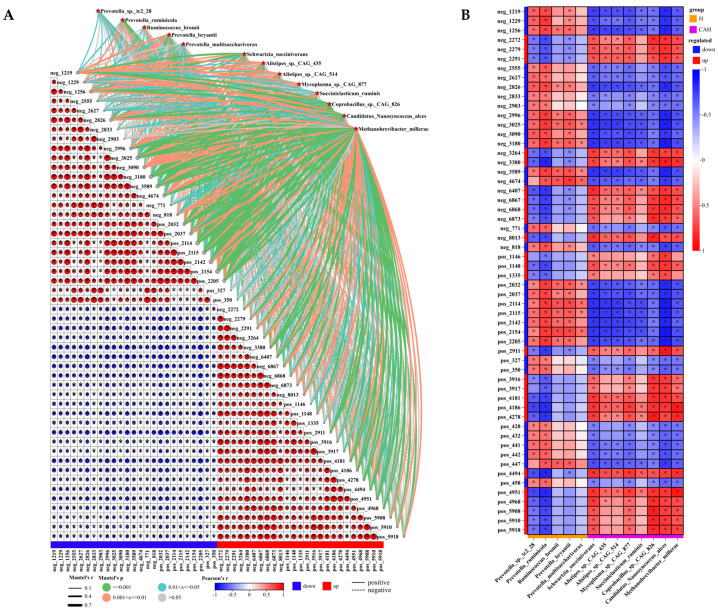
Correlation between rumen differential biomarkers and differential metabolites. (**A**) demonstrates Mantel’s r analysis of rumen differential biomarkers with differential metabolites. Spearman’s correlation coefficient is indicated by the colored circles. Edge width corresponds to the Mantel’s r statistic for the corresponding distance correlations, and edge color denotes the statistical significance based on permutations. (**B**) Exhibits Spearman’s correlation analysis of rumen differential biomarkers with differential metabolites. In the heat map, * indicates significant correlation, and values indicate correlation coefficients.

With VIP > 1.5, log2FC absolute value > 4, and *p* < 0.05 as the screening criteria for differentially accumulated metabolites, 56 metabolites (25 up-regulated and 31 down-regulated by CAH vs. Hu lambs) were identified from a total of 6119 metabolites. Subsequently, a correlation analysis was conducted between the microbial markers and the identified differential metabolites, with the results presented in Figure 4A,B. Overall, biomarkers have a significant impact on the differential metabolites. The strongest correlation was with pos_2154 (2-(4-Amino-1-isopropyl-1H-pyrazolo[3,4-d]pyrimidin-3-yl)-1H-indol-5-ol); in order, the top five were (2-(4-Amino-1-isopropyl-1H-pyrazolo[3,4-d]pyrimidin-3-yl)-1H-indol-5-ol), pos_2114 (Leucodopachrome), pos_2115 (2,4-DPD), pos_2142 (Gibberellin A39), and neg_3589 ((R)-N-Methylsalsolinol), respectively (Figure 4A). In addition, up-regulated and up-regulated differential metabolites showed generally positive correlations with each other (Figure 4A). Meanwhile, the down-regulated and down-regulated differential metabolites were basically positively correlated with each other (Figure 4A). However, up-regulated and down-regulated differential metabolites were basically negatively correlated with each other (Figure 4A). Correlation analysis of biomarkers with differential metabolites showed that rumen microbial markers in Hu sheep were generally positively correlated with down-regulated metabolites and negatively correlated with up-regulated metabolites, especially *Prevotella_sp._tc2_28*, *Prevotella_ruminicola*, and *Prevotella_bryantii* (Figure 4B). Additionally, CAH lamb rumen microbial markers overall showed negative correlations with down-regulated metabolites and positive correlations with up-regulated metabolites (Figure 4B).

## 4. Discussion

### 4.1. Effect of Three-Way Crosses on the Rumen Macrogenome of Sheep

#### 4.1.1. Effect of Three-Way Crossbreeding on Rumen Microbial Diversity in Sheep

Crossbreeding can exploit heterosis to allow the progeny to acquire the advantageous characteristics of diverse varieties, thus improving the growth performance of livestock and poultry. The findings of this preliminary experiment indicated that three-way-crossbred sheep CAH significantly altered rumen parameters and increased body weights. Additionally, 16S rRNA V3−V4 sequencing revealed that three-way hybridization elevated rumen microbial α-diversity, and the presence of rumen microbes in lambs was notably distinct between Hu and CAH lambs at 135 days of age [8]. However, microbial communities are subject to influences such as host genetics, sex, diet, age, environment, geographic location [11,44,45,46,47,48], and the vertical transmission of gut microbes [49]. Furthermore, the results of Wang et al. demonstrated that rumen microbes contributed 20% of the phenotypic variation when host genetics were considered [11]. Interestingly, our research has revealed a significant decrease in the rumen fluid VFA molar concentration in the three-way-hybrid lamb compared to the Hu lamb. However, the VFA molar ratio and acetic acid/propionic acid were not significantly impacted [8]. Hence, we hypothesized that hybridization may impact rumen fermentation and VFA utilization by modulating the composition and metabolism of rumen microbes. Species-level microorganism PCoA and NMDS analyses revealed that archaea, bacteria, and fungi were clearly segregated in the rumen of Hu and CAH lambs. Meanwhile, α-diversity assessment of rumen microorganisms showed that the ACE and Chao1 indices were significantly higher in CAH than in Hu lamb at the species, KEGG-enzyme, and CAZy-family levels. In addition, the Simpson and Shannon indices in the CAH group were significantly higher than in Hu sheep at the CAZy-family level. This result may be related to the fact that host genetics play a key role in determining rumen microbial communities. This result may be related to the fact that different gene expression programs and gene flow may influence microbial composition [26,50,51]. Meanwhile, the effect of hybridization on the offspring microbiota was greater than the maternal effect [50,52]. Moreover, the diversity of the rumen ecosystem was more beneficial for maintaining rumen homeostasis and resistance to environmental changes [53].

#### 4.1.2. Effect of Three-Way Crossbreeding on Rumen Microbial Composition in Sheep

Similar to many previous studies using metagenomics to assess the rumen microbiome, bacteria were the most abundant microbial kingdom in the rumen [54], accounting for more than 76.98% of the community. The experimental results show that, at the phylum level, the dominant bacteria remained Bacteroidetes and Firmicutes, which is consistent with the structure of the dominant rumen bacteria in ruminants (sheep [8,55], goat [48], cattle [56], dairy cows [57], and yak [58]). It was found that, at the top 30 abundance, three-way crossbreeding significantly increased Euryarchaeota (*Methanobrevibacter_millerae*), Tenericutes (*Mycoplasma_sp._CAG_877*), Candidatus Saccharibacteria (*Candidatus_Nanosyncoccus_alces*), Synergistetes, Planctomycetes, Lentisphaerae, Verrucomicrobia, Cyanobacteria, Chloroflexi, Acidobacteria, Candidatus Thermoplasmatota, Candidatus Absconditabacteria, and Candidatus Gracilibacteria but significantly reduced Bacteroidetes (*Prevotella_sp._tc2_28*, *Prevotella_ruminicola*, *Prevotella_sp._ne3005*, *Prevotella_brevis*, *Prevotella_bryantii*, *Prevotella_sp._BP1_148*, and *Prevotellaceae_bacterium_HUN156*) and Fusobacteria. Furthermore, at the species level, three-way crossbreeding also significantly increased Unclassified (*bacterium_F082*, *bacterium_P3*, *bacterium_F083*, *bacterium_P201*, *Bacteroidales_bacterium_WCE2008*, *Bacteroidales_bacterium_WCE2004*, and *Clostridiales_bacterium*) and Bacteroidetes (*Alistipes_sp._CAG_435* and *Alistipes_sp._CAG_51*) but decreased Firmicutes (*Ruminococcaceae_bacterium_P7*). The results indicate that the process of three-way hybridization has a notable impact on the microbial composition of the rumen. The study illustrated that Verrucomicrobiota, which is enriched with genes linked to the breakdown of lignocellulosic polymers and the fermentation of degradation byproducts into VFAs, along with Synergistota, enhanced the conversion of cellulose degradation into VFAs for organismal utilization [59,60,61]. Meanwhile, Euryarchaeota contain physiologically diverse groups of archaea, such as methanogens, extremophilic archaea, and hyperthermophilic archaea, for which methane production is an integral part of their metabolism [62]. The abundance of Tenericutes is closely related to the metabolic state. Studies in a mouse model of the metabolic syndrome found that the abundance of Bacteroidetes and Tenericutes was strongly associated with obesity-related inflammation [63]. Moreover, there was lower Tenericutes abundance in metabolically unhealthy, obese individuals [64] and individuals with reduced insulin sensitivity [65]. In addition, studies have shown that disease and inflammation can cause a decrease in the abundance of Acidobacteria [66,67,68] and that obesity increases Acidobacteria abundance [69]. Lentisphaerae abundance is reduced in disease [70] and immunodeficiency [71] and possesses a polysaccharide degradation function [72,73]. It is evident that rumen microbes and their hosts evolve synergistically during long-term adaptation to the environment and co-regulate host physiology [11,12,13,14,15]. The findings indicate that three-way crossings have the potential to modify microbial composition, thereby impacting rumen function and metabolic processes. Meanwhile, this experimental study found that three-way crossbreeding of the sheep rumen dominant genus *Prevotella* was significantly reduced, but at the phylum level, the three-way crossbreeding significantly reduced the abundance of Bacteroidetes and increased Firmicutes/Bacteroidetes (F/B). Studies have shown that *Prevotellaceae* produce mainly acetate and succinate [74] and are negatively correlated with growth rates [75] and production performance [76,77] in ruminants. However, this is also contrary to some studies that found *Prevotella* species to be associated with higher milk production [78,79]. Meanwhile, studies have shown that lower F/B is strongly associated with weight loss [80], reduced feed efficiency [18], and reduced milk fat production in dairy cows [77]. This result suggests that, while focusing on the dominant bacteria playing an important role in the physiological functions of the host, even more noteworthy are the low-abundance bacteria. Despite their low abundance, their taxa numbers and diversity are much higher than those of the host’s high-abundance bacteria and may play a key role in various aspects of host physiology. Furthermore, in complex rumen ecosystems, microbiota interactions may be more important for ecosystem function than abundance [75,81]. The Mantel test allows for a visual understanding of the relationship between community matrices and environmental factors. The Mantel test found that rumen microorganisms significantly affected body size indexes (BW and TC), rumen enzyme activities (xylanase, amylase, and CMC), VFA molar concentration (PA, IBA, BA, IVA, VA, and TVFAs), and VFA molar proportion (PAR and VAR), with the highest correlation with PA. The findings indicated that the microorganisms in the rumen of sheep exhibit a high level of complexity and diversity. Moreover, the construction of species-level correlation network diagrams revealed significant correlations among microbial communities. Consequently, alterations in the gastric microbiome of the crossbred offspring could have been impacted by animal genetics. Previous research has indicated that gene flow can influence microbial composition, with this trait being heritable. Moreover, hybridization was found to have a more pronounced impact on microbial composition compared to maternal effects, as supported by related studies [11,26,50,52,53,75,82].

#### 4.1.3. Effect of Three-Way Crossbreeding on Rumen Microbial Functions in Sheep

Carbohydrates can significantly influence microbiota structure and microbial interactions, whether competitive or synergistic. The evolution and acquisition of specific CAZymes provide a competitive advantage for certain bacteria. Additionally, microbial carbohydrate degradation activities, including glycoside hydrolases (GH), polysaccharide lyases (PL), carbohydrate esterases (CE), and auxiliary activity (AA), provide nutrients to the host. Typically, CAZymes are accompanied by carbohydrate-binding modules (CBMs), which are crucial for enhancing the catalytic activity of the attached CAZyme but do not possess catalytic activity themselves [36]. Furthermore, microorganisms produce CAZymes that work in synergy with the animal body to break down complex carbohydrates before being further metabolized and utilized by the organism, generating new bio-signaling molecules that act as a bridge between host and microbial regulation. Macrogenomic analysis revealed that the GH and GT families were enriched in rumen species, which is similar to previous findings [83,84]. Meanwhile, a significant correlation between the CAZy family was found by constructing a CAZy-family level correlation network diagram and differential correlation abundance top five for GT2 (Hexosyltransferases), GH43 and GH28 (hydrolyse O- and S-glycosyl compounds), GH1 (Hexosyltransferases, hydrolyse O- and S-glycosyl compounds), and GT51 (peptidoglycan glycosyltransferase). Among them, the GH family is mainly involved in cellulases, hemicellulose, oligosaccharide-degrading enzymes, and related debranching enzymes [84,85]. It was found that crossbred sheep (CAH) were significantly enriched in Hexosyltransferases and hydrolyse O- and S-glycosyl compounds (GH13, GH3, and GH1), hydrolyse O- and S-glycosyl compounds (GH28), Hexosyltransferases and Pentosyltransferases (GT1), and hydrolyse O- and S-glycosyl compounds (GH38, GH92), while Hu sheep were significantly enriched in Hexosyltransferases (GT2), hydrolyse O- and S-glycosyl compounds and peptidoglycan lytic transglycosylase (GH23), hydrolyse O- and S-glycosyl compounds (GH2, GH43, and GH73), Hexosyltransferases and hydrolyse O- and S-glycosyl compounds (GH35), and GT0 (Glycosyltransferases not yet assigned to a family). In addition, in the GH family, GH13 had the highest abundance, with GH23 and GH3 second and third, respectively. In the GT family, GT2 had the highest abundance, with GT51 and GT28 second and third, respectively. It was found that in the top 30, differential function in the rumen of CAH and Hu lambs was mainly enriched in GH and GT but had essentially no significant effect in CBMs, CEs, AAs, and PLs. This result may be related to the fact that in our previous study, we found that there were significant differences in VFA molar content between three-way-crossbred lambs and Hu, but basically no significant changes in VFA molar proportion [8]. Additionally, the rumen VFA molar proportion remained essentially unchanged, which may be related to the fact that ruminants can maintain the stability of rumen molar proportion through a complex regulatory mechanism [86]. Studies have shown that an increase in VFA molar concentration can enhance pancreatic sensitivity, leading to increased insulin secretion, increased fat oxidation, and decreased fat deposition and body weight [87]. Furthermore, studies have indicated that an increase in VFA concentration can increase anorexigenic signaling and decrease orexigenic signaling, affecting food intake, resulting in negative energy balance and influencing feed intake [88]. The pre-fasting VFAs molar concentration of Hu sheep was significantly higher than that of CAH, while the body weight was lower than that of CAH. This result is consistent with the theory mentioned above [87] and further demonstrates the difference in the efficiency of VFA utilization between the bodies of Hu sheep and crossbred progeny. Macrogenomic KEGG-enzyme analysis revealed that CAH significantly increased Oxidoreductases (pyruvate-ferredoxin/flavodoxin oxidoreductase), Transferases (DNA-directed RNA polymerase, pyruvate, and phosphate dikinase), Hydrolases (catalyzing transmembrane movement of substances, cellular and subcellular movement, and Carboxylic ester hydrolases), Isomerases (DNA topoisomerase-ATP-hydrolysing), and Ligases (long-chain-fatty-acid-CoA ligase, phenylalanine-tRNA ligase, and isoleucine-tRNA ligase) but decreased Oxidoreductases (NADH:ubiquinone reductase-H^+^-translocating and glutamate synthase), Transferases (site-specific DNA-methyltransferase), Hydrolases (type I site-specific deoxyribonuclease and beta-galactosidase), and Isomerases (peptidylprolyl isomerase). Meanwhile, a significant correlation between enzymes was found by constructing a KEGG-enzyme level correlation network diagram. The differential correlation abundance top five included DNA-directed RNA polymerase, DNA topoisomerase-ATP-hydrolysing, peptidylprolyl isomerase, cellular and subcellular movement, and NADH: ubiquinone reductase -Na^+^-transporting. Therefore, it was speculated that three-way hybridization could modulate rumen metabolite production by altering rumen microbial composition and thus enriching the KEGG enzyme and CAZy family.

### 4.2. Effect of Three-Way Crossbreeding on Rumen Metabolism in Sheep

Ruminal metabolites are the basis for participation in the physiological metabolism of ruminants and bridge the interaction between rumen microorganisms and the host [75,89]. The current experimental study revealed notable variances in rumen metabolites between CAH and Hu sheep. The analysis of the KEGG pathway enriched by the top five differential metabolites indicated that a majority of the differential metabolites are involved in tyrosine metabolism; valine, leucine, and isoleucine biosynthesis; and chemical carcinogenesis-DNA adducts; and that the riboflavin metabolism pathways were significantly down-regulated in CAH. Only Succinic acid, 5,6-Indolequinone-2-carboxylic acid, Maleic acid, Vapreotide, 4-(Methylnitrosamino)-1-(3- pyridyl)-1-butanol glucuronide, riboflavin, and reduced riboflavin were significantly up-regulated in the CAH lamb. However, differential metabolites in the porphyrin metabolism pathway were largely significantly up-regulated in CAH, with only D-Urobilinogen, Porphobilinogen, (3b,7b,22x)-Cucurbita-5,24-diene-3,7,23-triol 7- glucoside, and primary fluorescent chlorophyll catabolite being down-regulated. Subsequently, our analysis of rumen microbes and KEGG pathway differential metabolites using the Mantel test revealed that the rumen microbes were most strongly associated with neg_1108 (Succinic acid), followed by pos_3963 (Urobilinogen) and neg_998 (Dopaquinone) in second and third order, respectively. In this study, we observed a significant reduction in 2-oxoglutarate in CAH rumen fluid. Of particular note, succinate, a metabolite closely associated with rumen microbes, plays a crucial role in the organismal energy metabolism as a downstream product of the host cell and gut microbial metabolite 2-oxoglutarate dehydrogenase complex. Previous research has shown that succinate serves as an intermediate metabolite in the Krebs cycle, participating in inflammatory responses via endocrine and paracrine signaling pathways, facilitating skeletal muscle protein synthesis, regulating myofibrillar restructuring, contributing to energy supply, and maintaining glucose homeostasis [90,91]. Furthermore, succinate functions as an epigenetic regulator involved in gene transcription, translation, and post-translational modification [92]. Hormones (e.g., IGF-1) and nutrients (e.g., amino acids) are generally essential in the regulation of skeletal muscle protein synthesis. Succinate further stimulates skeletal muscle protein synthesis through the Erk/Akt signaling pathway by activating the Akt/mTOR/S6 cascade and inhibiting FoxO3a [93]. It is worth noting that certain amino acids and TCA cycle intermediates, such as α-ketoisocaproate, β-hydroxy-β-methylbutyrate, and 2-oxoglutarate, have also been identified to promote skeletal muscle growth by increasing protein synthesis and inhibiting protein degradation. Meanwhile, metabolite analysis by the succinate and KEGG top five pathways showed that succinate had a significant negative correlation with a large number of metabolites and significantly positively correlated with only a few metabolites (Maleic acid, Riboflavin, Reduced riboflavin, 8-Ethyl-12-methyl-3-vinylbacteriochlorophyllided, Red chlorophyll catabolite, 12-Ethyl-8-propylbacteriochlorophyllided, Urobilinogen, and 5-Oxo-delta-bilirubin). Then, we further analyzed the correlation analysis of succinic acid with species-level microbial markers and found that succinic acid showed a negative correlation with Hu lamb species-level markers (*Prevotella_sp._tc2_28*, *Prevotella_ruminicola*, and *Prevotella_bryantii*), while it showed a positive correlation with CAH lamb species-level markers (*Schwartzia_succinivorans*, *Alistipes_sp._CAG_435*, *Alistipes_sp._CAG_514*, *Mycoplasma_sp._CAG_877*, *Succiniclasticum_ruminis*, *Coprobacillus_sp._CAG_826*, *Candidatus_ Nanosyncoccus_alces* and *Methanobrevibacter_millerae*).

In addition, we collected rumen fluid non-target metabolomic data and phenotypic data from Hu and CAH lambs with highly uniform feeding environments for correlation analysis. Our study demonstrated that the rumen metabolites significantly affected body size indexes (body weight, chest circumference, and tube circumference), rumen enzyme activities (MCC, xylanase, amylase, and CMC), pH, VFAs molar concentration (propionic acid, butyric acid, isobutyric acid, valeric acid, and TVFA), and VFA molar proportion (propionic acid ratio, isobutyric acid ratio, and valeric acid ratio). Mantel analysis of biomarkers and differential metabolites of rumen microorganisms at species level showed that biomarkers significantly affected differential metabolites and that differential metabolites were significantly correlated with each other. However, the interactions between microbial metabolites and host physiology are complex and are influenced by the environment, microbes, and host genetics, and act as signaling molecules and substrates for metabolic reactions in the host [93]. In conclusion, three-way hybridization may regulate succinate metabolism and promote lamb growth and development by affecting the rumen microbiota. Our present research indicates a close relationship between rumen metagenomics and metabolomics with host traits. Furthermore, it was observed that the rumen microbes and metabolites have the ability to interact with each other, collectively influencing host phenotypes.

## 5. Conclusions

This study identified notable variances in the taxonomic characteristics, functions, and metabolites of rumen microorganisms in Hu and CAH lambs. The diversity of offspring microbial communities was significantly increased through three-way crossbreeding, with biomarkers playing a crucial role in influencing rumen differential metabolites. Additionally, microbes significantly affected the top five pathway metabolites, with a specific focus on succinic acid. This study’s findings indicated notable interactions between rumen microbes and metabolites. Despite controlling for various external factors such as feed and management practices that could affect lamb growth, the study revealed that three-way crossbreeding can lead to alterations in rumen metabolite composition, microbial composition and function, and a significant association with host phenotype.

## Data Availability

Follow-up research on this project is ongoing; please contact the corresponding author with reasonable requests. All data covered in the article are available from the authors.
